# 

**Published:** 1919-08-02

**Authors:** 


### The Book Market.

The Macmillan Co.: "The Newer Knowledge of Nutrition." By E. V. McCollan. 6s. 6d. net.

Longmans, Green & Co.: "Influenza." By Sir Arthur Newsholme, K.C.B., M.D. 3s. 6d. net. Wm. Heinemann.

Longmans, Green & Co.: "Anæsthesia." By A. D. Prenderville. 3s. 6d. net.

J. Wright and Sons, Ltd.: "The Medical Annual." 1919. 20s. net.

H. K. Lewis: "Massage and the Swedish Movements." By K. W. Ostrom, Eighth Edition. 5s. net.

Cassell and Co.: "Infant and Young Child Welfare." By Harold Scurfield, M.D. 5s. net. "The Story of English Public Health." By Sir Malcolm Morris, K.C.V.O. 5s. net.

J. Wright & Sons, Ltd., Bristol: "Gunshot Injuries to the Blood Vessels." By George H. Makins, G.C.M.G., C.B. 21s. net.

J. & A. Churchill: "Vicious Circles in Disease." By Jamieson P. Hurry, M.A., M.D. 15s. net.

Cassell & Co.: "Food and the Public Health." By W. G. Savage, B.Sc., M.D., etc. 5s. net.

Cassell & Co.: "Housing and the Public Health." By John Robertson, M.D. 5s. net.

### Gifts and Bequests.

Mr. George Wyman has left £1,000 to the Peterborough Infirmary.

The Children's Hospital, Birmingham, benefits by a legacy of £100 under the will of the late Mr. E. D. Beesley, of Handsworth.

The Southwell and District Nursing Association received a legacy of £100 under the will of the Rev. William Glaister, of Southwell, Nottingham.

Mr. J. H. Pearce, of Aylbourton, Glos., bequeathed £500 to Lydney Cottage Hospital and one half of the residue of his estate between Pershole Hospital and Worcester Infirmary.

Mr. Montague Blaker, of Lewes, has left £150 for charitable purposes.

Mr. G. D. Stelfox has bequeathed £100 to Altrincham Dispensary, and £50 to Noble's Hospital, Douglas.

Dr. H. Wilde, of Alderley Edge, an expert in electricity and mining, has left £100 each to the Royal Lancashire Asylum for Idiots and Henshaw's Blind Asylum.

The Great Northern General Hospital has received from the Richard Cloudesley Trustees £705 (an increase of £60 on last year's grant) and £100 from the League of the Roses (per Miss M. F. Roby, Chairman), making the League's contribution £700 since January.

### Hospital and Institutional Results.

**The Hostel of God, Clapham Common, S.W.**— The hostel has in an annexe twelve separate bedrooms for the treatment of severe cases of illness, or for those which require operation. The patients are all drawn from outside, and are attended by their own medical men. The hostel received £2,083 from legacies last year.

**The Florence Nightingale Hospital for Gentlewomen, Lisson Grove, N.W.**—Of the 425 patients admitted during 1918, 102 were related to officers in the Navy, Army, and Air Force. The fees contributed by the patients, who chiefly depend upon fixed incomes, have not been raised.

**St. George's Hospital.**—This is one of the first full annual reports that has reached us. The average weekly cost of each in-patient during 1918 was £3 5s. 7d. This high rate would be reduced if the beds were more fully occupied. We notice that of the 341 beds only 284 were on an average occupied during 1918. No doubt the reservation of some wards for soldiers kept the average low.

**Crichton Royal Institution, Dumfries.** — The medical officer notices "a distinct increase in the proportion of cases attributed to such factors as emotional stress (worry, anxiety, grief, etc.), overwork, and bodily ill-health, factors which may probably be co-related, and largely attributed to the prolonged stress of the war." The institution is for mental disease.

**Glasgow Ophthalmic Institution.** — The annual report is not sent to subscribers unless they ask for it. When the statistics for the previous year have been prepared a card, giving the salient features, is distributed, and this also serves to remind supporters that annual subscriptions are due.

**Essex County Hospital.** — The accounts show a balance for 1918 of £1,084 on the wrong side, but they include £964 spent on emergency marquees for wounded soldiers, on whose behalf during the year a sum of £16,645 was paid by the War Office.

**Hampstead General Hospital.**—The Council have entered into an arrangement with the Hampstead Borough Council whereby the hospital will be linked with the tuberculosis department of the Public Health Committee so as to afford special facilities to the department in the treatment of persons suffering from tuberculosis.

**The Royal Hospital for Diseases of the Chest.**— The annual report is complete with the exception of the lists of subscribers. It is hoped that the hospital will shortly again be available for civilian use. The special appeal of last year produced £2,358 and the income altogether exceeded the expenditure by £1,966.

**The Coleraine Cottage Hospital.**—This hospital of seventeen beds and cots is in connection with the District Nursing Association and affiliated to Queen Victoria Jubilee Institute for Nurses. The financial position, which is satisfactory, improved during the year.

**The General Hospital, Birmingham.**—The average weekly cost of each in-patient was £3 2s. 7d. The hospital received last year £40,284 and spent £58,454, but some donations and legacies, amounting to £2,449, are carried direct to investment account. Subscriptions have increased from £7,362 in 1916 to £9,692 in 1919.

**Eversfield Chest Hospital, St. Leonards-on-Sea.**— The report this year is, as usual, an attractive one, in which the work is excellently illustrated and described. The mortgage on the building has been reduced from £4,500 to £2,500.

**Guy's Hospital, Natal.**—The annual report for 1918 has just been published, and the statistical returns show an increase in the number of admissions during the year, the average daily total being 123, as compared with 101 in the previous year. Large numbers of military cases were admitted during the first 4 months of the year, and this, in addition to the influenza epidemic which occurred in October and November last, accounts mainly for this increase. A new double-storied block of wards for Europeans to contain 60 beds is under construction and is to be ready shortly. The ravages caused by the terrible epidemic of Spanish influenza were keenly felt. 548 causes were treated and 71 deaths occurred from this cause, which brought the mortality returns up to an unprecedentedly high rate (30 to 40 of the nursing staff were stricken, but they all recovered). Other statistics were as follows: 40 cases of enteric fever, 3 died; 19 cases of phthisis, 11 died; 15 cases of dysentery, 2 died; 196 cases of malaria, 1, admitted in a moribund condition, died.

### Buildings Contemplated and in Progress.

Middleton.—The West Riding County Council has decided to provide fifty more beds at the Middleton Sanatorium at a cost of £10,300.

Nelson.—It has been decided to erect a new hospital, at a cost of £40,000, as a war memorial. The Mayor has received an anonymous gift of £10,000 towards the object.

Redcar.—It has been decided to provide, without delay, a more commodious infectious diseases hospital.

Retford.—It has been decided that part of the war memorial shall take the form of a special wing to the new Retford Hospital, for which a site has been presented.

St. Ives.—The Town Council has passed plans, submitted by the directors of the Hain Steamship Co., for the conversion of Albany House into a cottage hospital.

Selby.—It is proposed to build a general hospital as a war memorial, the institution to take the place of the present cottage hospital.

Towyn.—The Towyn and Aberdovey Urban District Council has applied to the Red Cross Society for a grant of £1,000 in connection with the Society's scheme for establishing cottage hospitals in Merioneth out of Red Cross funds and effects on demobilisation.

Shrewsbury.—The Atcham and Shrewsbury Councils are joining in a scheme for the provision of isolation hospital accommodation to serve both districts.

Tottenham.—It is proposed to enlarge the Prince of Wales's Hospital at an estimated cost of £146,000.

Willian.—The Hertfordshire County Council has decided to erect a county sanatorium at Willian at an estimated cost of £67,124.

Worksop.—It has been decided to erect, as part of the town's war memorial, an additional administrative block and new male ward at the Worksop Victoria Hospital.

Atherstone.—The local war memorial will take the form of a cottage hospital. A gift of £500 has been promised towards the cost.

Bradford.—The Corporation Health Committee has under consideration the question of providing a municipal hospital.

Colchester.—Essex County Hospital is to be enlarged, as a local war memorial.

Scunthorpe.—The executors of the late Lord St. Oswald have given 14½ acres of land as a site for the war memorial general hospital.

### MATRONS' AND SISTERS' APPOINTMENTS.

BOROUGH OF HORNSEY.—Miss L. E. Snape has been appointed health visitor. She was trained at the Northern Hospital, Liverpool, and has been health visitor under the Leicester County Council.

CARDIGAN SANATORIUM, NEAR WAKEFIELD.—Miss Alice M. Anthony has been appointed sister. She was trained at Rochdale Union Infirmary, has since been ward sister at the Queen's Park Military Hospital, and sister-in-charge of the Blackburn and Commonside Sanatoria.

EAST CLIFF HOUSE, CLIFTONVILLE, MARGATE.—Miss F. M. Punter has been appointed matron. She was trained at the Royal Infirmary, Gloucester, and is at present assistant matron and home sister at the Alexandra Hospital for Children with Hip Disease, Queen Square, W.C., where she has also had entire charge of housekeeping and has been acting matron for eleven months. Miss Punter has been sister at the Isolation Hospital, Gloucester, the Royal Victoria Hospital for Consumption, Edinburgh, the Welsh Metropolitan War Hospital, Cardiff; night sister at the Devonshire Hospital, Buxton; assistant matron and housekeeper at the County Sanatorium, Bovey Tracey; and matron of the Exeter Tuberculosis Sanatorium.

GENERAL HOSPITAL, NEWRY, CO. DOWN.—Miss Bridie McPolin has been appointed matron. She was trained at the Mater Misericordiæ Hospital, Dublin, where she was later masseuse, and has been sister at the above-mentioned hospital.

HIGH TEAMS HOSPITAL, GATESHEAD.—Miss Lily B. M. Hall has been appointed matron. She was trained at Queen Mary's Hospital, Stratford, and at the North Western Fever Hospital. She has been superintendent nurse at Sudbury Infirmary, Suffolk, health visitor under the Durham County Council, matron of Sealburn Hospital, Ryton, and acting matron of Holywood Hall, simultaneously, under the Durham County Council, temporary acting matron of Clift House Sanatorium, Bristol, and matron of Tredegar Infirmary, Mon. Miss Hall has also done military nursing, and she holds the certificates of the Central Midwives Board and the Royal Sanitary Institute.

INFIRMARY AND DISPENSARY, PETERBOROUGH.—Miss Kathleen T. Browne has been appointed matron. She was trained at St. Bartholomew's Hospital, Rochester, and has been matron of the Torbay Hospital, Torquay.

ISOLATION HOSPITAL, BEAUFORT.—Miss Winifred James has been appointed matron. She was trained at Kettering General Hospital and at the Auckland and Sheldon Joint Hospital, Durham, where she was later staff nurse. She has also been district nurse at Choppington, Northumberland, school nurse under the Luton Education Committee, matron of the Stanhope and Weardale Infectious Hospital, Durham, and temporary matron of the Dunstable District Hospital.

QUEEN ALEXANDRA'S MILITARY NURSING SERVICE FOR INDIA.—Miss E. M. Cott has been appointed to the above Service. She was trained at the London Hospital.

R.A.F. HOSPITAL, SWANAGE, DORSET.—Miss Margaret Webster has been appointed sister. She was trained at the Metropolitan Hospital, London, and has been sister at the military hospital attached, and also sister at the Grata Quies Hospital, Bournemouth.

ROYAL SOUTH HANTS AND SOUTHAMPTON HOSPITAL.—Miss Jenkins, A.R.R.C., has been appointed matron in succession to Miss E. B. Harradine. Miss Jenkins was trained at Guy's Hospital, and for three years before the war was assistant matron at the Royal South Hants and Southampton Hospital, and since August 1914 has been on active service in France. She holds the certificates of the Central Midwives Board and of the Incorporated Society of Trained Masseuses.

URBAN DISTRICT COUNCIL OF DARTFORD.—Miss A. S. Hiscocks has been appointed health visitor. She was trained at the Bethnal Green Infirmary, and has been health visitor under the Devon County Council, district nurse, health visitor, and school nurse at Hatfield.

